# Glioblastoma: State of the Art and Future Perspectives

**DOI:** 10.3390/cancers11081091

**Published:** 2019-07-31

**Authors:** Ghazaleh Tabatabai, Hiroaki Wakimoto

**Affiliations:** 1 Interdisciplinary Division of Neuro-Oncology, Hertie Institute for Clinical Brain Research, Center for Neuro-Oncology, Comprehensive Cancer Center Tübingen Stuttgart, University Hospital Tübingen, Eberhard Karls University Tübingen, 72076 Tübingen, Germany; 2 Department of Neurosurgery, Massachusetts General Hospital, Harvard Medical School Boston, Boston, MA 02114, USA

This special issue is dedicated to glioblastoma and elucidates this disease from different perspectives. Despite multimodal therapies, the prognosis is still dismal. Many features contribute to this therapeutic challenge including high intratumoral and intertumoral heterogeneity, resistance to therapy, migration and invasion, and immunosuppression. With the advent of novel high throughput technologies, significant progress has been made to understand molecular and immunological signatures underlying the pathology of glioblastoma. This special issue aimed at updating researchers on current topics and progress made in basic, preclinical, and clinical glioblastoma research.

The original articles in this special issue present novel findings on molecular mechanisms of radiosensitization, radioresistance and acquired resistance to therapy (Kessler et al., Müller-Längle et al., Shah et al., Llaguno-Munive et al.) [1,2,3,4], cell migration (Nowicki et al., Hübner et al.) [5,6], intracellular drug levels (Colin et al.) [7], strategies targeting renewal capacities of cancer stem-like cells (Gravina et al., Sansalone et al., Linder et al.) [8,9,10], mathematic modeling of synergy, machine learning, deep learning (Kim et al., Hana et al., Wong et al.) [11,12,13], distinct signaling pathways (Barbagallo et al., Akgül et al., Bensalma et al., Saito et al., Liu et al.) [14,15,16,17,18], prognostic and predictive effects of imaging patterns (Jungk et al., Puig et al.) [19,20], tumor-associated epilepsy (Berendsen et al.) [21], and novel models and experimental therapeutic approaches (Berthier et al., Offenhäuser et al., Privat-Maldonado et al., Lozada-Delgado et al.) [22,23,24,25]. 

Moreover, review articles summarize the current state of knowledge in the fields of platelets (Marx et al.) [26], subventricular zone (Altmann et al.) [27] and microenvironment (Schiffer et al.) [28], lentiviral vectors (Del Vecchio et al.) [29], allergic inflammation (Costanza and Finocchiaro) [30], elderly patients (Minniti et al.) [31], EMT and authophagy (Colella et al.) [32], notch signaling and CXCL12 signaling (Bazzoni et al., Giordano et al.) [33,34], protein phosphatases (Tomiyama et al.) [35], nitric oxide antagonism (Fahey and Girotti) [36], virus-based immunotherapy (Martikainen and Essand; Stepanenko et al.) [37,38], tumor-treating fields (Fabian et al.) [39], amino acid PET (Lohmann et al.) [40], cholesterol metabolism (Ahmad et al.) [41], preclinical modeling (Rominiyi et al.) [42], and EGFR as a therapeutic target (Gao et al.) [43].

It becomes clear that a holistic view from different angles is required to understand this complex disease and discover novel therapeutic targets and biomarkers. We are grateful for all the work the authors have included in this special issue.

Finally, we would like to emphasize the most important perspective, i.e., our patients’ perspectives. From their point of view, the main readout for success in patient-centered research is prolonged survival with maintained quality of life. A culture of continued collaboration between disciplines and research teams will be necessary to meet this challenge.

## References

[1] Kessler J., Hohmann T., Güttler A., Petrenko M., Ostheimer C., Hohmann U., Bache M., Dehghani F., Vordermark D. (2019). Radiosensitization and a Less Aggressive Phenotype of Human Malignant Glioma Cells Expressing Isocitrate Dehydrogenase 1 (IDH1) Mutant Protein: Dissecting the Mechanisms. Cancers.

[2] Müller-Längle A., Lutz H., Hehlgans S., Rödel F., Rau K., Laube B. (2019). NMDA Receptor-Mediated Signaling Pathways Enhance Radiation Resistance, Survival and Migration in Glioblastoma Cells—A Potential Target for Adjuvant Radiotherapy. Cancers.

[3] Shah S., Rodriguez G., Musick A., Walters W., de Cordoba N., Barbarite E., Marlow M., Marples B., Prince J., Komotar R. (2019). Targeting Glioblastoma Stem Cells with 2-Deoxy-D-Glucose (2-DG) Potentiates Radiation-Induced Unfolded Protein Response (UPR). Cancers.

[4] Llaguno-Munive M., Romero-Piña M., Serrano-Bello J., Medina L., Uribe-Uribe N., Salazar A., Rodríguez-Dorantes M., Garcia-Lopez P. (2019). Mifepristone Overcomes Tumor Resistance to Temozolomide Associated with DNA Damage Repair and Apoptosis in an Orthotopic Model of Glioblastoma. Cancers.

[5] Nowicki M., Hayes J., Chiocca E., Lawler S. (2019). Proteomic Analysis Implicates Vimentin in Glioblastoma Cell Migration. Cancers.

[6] Hübner M., Hinske C., Effinger D., Wu T., Thon N., Kreth F., Kreth S. (2018). Intronic miR-744 Inhibits Glioblastoma Migration by Functionally Antagonizing Its Host Gene MAP2K4. Cancers.

[7] Colin M., Delporte C., Janky R., Lechon A., Renard G., Van Antwerpen P., Maltese W., Mathieu V. (2019). Dysregulation of Macropinocytosis Processes in Glioblastomas May Be Exploited to Increase Intracellular Anti-Cancer Drug Levels: The Example of Temozolomide. Cancers.

[8] Gravina G., Mancini A., Colapietro A., Delle Monache S., Sferra R., Vitale F., Cristiano L., Martellucci S., Marampon F., Mattei V. (2019). The Small Molecule Ephrin Receptor Inhibitor, GLPG1790, Reduces Renewal Capabilities of Cancer Stem Cells, Showing Anti-Tumour Efficacy on Preclinical Glioblastoma Models. Cancers.

[9] Sansalone L., Veliz E., Myrthil N., Stathias V., Walters W., Torrens I., Schürer S., Vanni S., Leblanc R., Graham R. (2019). Novel Curcumin Inspired Bis-Chalcone Promotes Endoplasmic Reticulum Stress and Glioblastoma Neurosphere Cell Death. Cancers.

[10] Linder B., Wehle A., Hehlgans S., Bonn F., Dikic I., Rödel F., Seifert V., Kögel D. (2019). Arsenic Trioxide and (−)-Gossypol Synergistically Target Glioma Stem-Like Cells via Inhibition of Hedgehog and Notch Signaling. Cancers.

[11] Kim Y., Lee J., Lee D., Othmer H. (2019). Synergistic Effects of Bortezomib-OV Therapy and Anti-Invasive Strategies in Glioblastoma: A Mathematical Model. Cancers.

[12] Hana T., Tanaka S., Nejo T., Takahashi S., Kitagawa Y., Koike T., Nomura M., Takayanagi S., Saito N. (2019). Mining-Guided Machine Learning Analyses Revealed the Latest Trends in Neuro-Oncology. Cancers.

[13] Wong K., Rostomily R., Wong S. (2019). Prognostic Gene Discovery in Glioblastoma Patients using Deep Learning. Cancers.

[14] Barbagallo D., Caponnetto A., Brex D., Mirabella F., Barbagallo C., Lauretta G., Morrone A., Certo F., Broggi G., Caltabiano R. (2019). CircSMARCA5 Regulates VEGFA mRNA Splicing and Angiogenesis in Glioblastoma Multiforme Through the Binding of SRSF1. Cancers.

[15] Akgül S., Patch A., D’Souza R., Mukhopadhyay P., Nones K., Kempe S., Kazakoff S., Jeffree R., Stringer B., Pearson J. (2019). Intratumoural Heterogeneity Underlies Distinct Therapy Responses and Treatment Resistance in Glioblastoma. Cancers.

[16] Bensalma S., Turpault S., Balandre A., De Boisvilliers M., Gaillard A., Chadéneau C., Muller J. (2019). PKA at a Cross-Road of Signaling Pathways Involved in the Regulation of Glioblastoma Migration and Invasion by the Neuropeptides VIP and PACAP. Cancers.

[17] Saito N., Hirai N., Aoki K., Suzuki R., Fujita S., Nakayama H., Hayashi M., Ito K., Sakurai T., Iwabuchi S. (2019). The Oncogene Addiction Switch from NOTCH to PI3K Requires Simultaneous Targeting of NOTCH and PI3K Pathway Inhibition in Glioblastoma. Cancers.

[18] Liu H., Su Y., Bamodu O., Hueng D., Lee W., Huang C., Deng L., Hsiao M., Chien M., Yeh C. (2018). The Disruption of the β-Catenin/TCF-1/STAT3 Signaling Axis by 4-Acetylantroquinonol B Inhibits the Tumorigenesis and Cancer Stem-Cell-Like Properties of Glioblastoma Cells, In Vitro and In Vivo. Cancers.

[19] Jungk C., Warta R., Mock A., Friauf S., Hug B., Capper D., Abdollahi A., Debus J., Bendszus M., von Deimling A. (2019). Location-Dependent Patient Outcome and Recurrence Patterns in IDH1-Wildtype Glioblastoma. Cancers.

[20] Puig J., Biarnés C., Daunis-i-Estadella P., Blasco G., Gimeno A., Essig M., Balaña C., Alberich-Bayarri A., Jimenez-Pastor A., Camacho E. (2019). Macrovascular Networks on Contrast-Enhanced Magnetic Resonance Imaging Improves Survival Prediction in Newly Diagnosed Glioblastoma. Cancers.

[21] Berendsen S., Spliet W., Geurts M., Van Hecke W., Seute T., Snijders T., Bours V., Bell E., Chakravarti A., Robe P. (2019). Epilepsy Associates with Decreased HIF-1α/STAT5b Signaling in Glioblastoma. Cancers.

[22] Berthier S., Larrouquère L., Champelovier P., Col E., Lefebvre C., Cottet-Rouselle C., Arnaud J., Garrel C., Laporte F., Boutonnat J. (2019). A New Patient-Derived Metastatic Glioblastoma Cell Line: Characterisation and Response to Sodium Selenite Anticancer Agent. Cancers.

[23] Offenhäuser C., Al-Ejeh F., Puttick S., Ensbey K., Bruce Z., Jamieson P., Smith F., Stringer B., Carrington B., Fuchs A. (2018). EphA3 Pay-Loaded Antibody Therapeutics for the Treatment of Glioblastoma. Cancers.

[24] Privat-Maldonado A., Gorbanev Y., Dewilde S., Smits E., Bogaerts A. (2018). Reduction of Human Glioblastoma Spheroids Using Cold Atmospheric Plasma: The Combined Effect of Short- and Long-Lived Reactive Species. Cancers.

[25] Lozada-Delgado E., Grafals-Ruiz N., Miranda-Román M., Santana-Rivera Y., Valiyeva F., Rivera-Díaz M., Marcos-Martínez M., Vivas-Mejía P. (2018). Targeting MicroRNA-143 Leads to Inhibition of Glioblastoma Tumor Progression. Cancers.

[26] Marx S., Xiao Y., Baschin M., Splittstöhser M., Altmann R., Moritz E., Jedlitschky G., Bien-Möller S., Schroeder H., Rauch B. (2019). The Role of Platelets in Cancer Pathophysiology: Focus on Malignant Glioma. Cancers.

[27] Altmann C., Keller S., Schmidt M. (2019). The Role of SVZ Stem Cells in Glioblastoma. Cancers.

[28] Schiffer D., Annovazzi L., Casalone C., Corona C., Mellai M. (2019). Glioblastoma: Microenvironment and Niche Concept. Cancers.

[29] Del Vecchio C., Calistri A., Parolin C., Mucignat-Caretta C. (2019). Lentiviral Vectors as Tools for the Study and Treatment of Glioblastoma. Cancers.

[30] Costanza M., Finocchiaro G. (2019). Allergic Signs in Glioma Pathology: Current Knowledge and Future Perspectives. Cancers.

[31] Minniti G., Lombardi G., Paolini S. (2019). Glioblastoma in Elderly Patients: Current Management and Future Perspectives. Cancers.

[32] Colella B., Faienza F., Di Bartolomeo S. (2019). EMT Regulation by Autophagy: A New Perspective in Glioblastoma Biology. Cancers.

[33] Bazzoni R., Bentivegna A. (2019). Role of Notch Signaling Pathway in Glioblastoma Pathogenesis. Cancers.

[34] Giordano F., Link B., Glas M., Herrlinger U., Wenz F., Umansky V., Brown J., Herskind C. (2019). Targeting the Post-Irradiation Tumor Microenvironment in Glioblastoma via Inhibition of CXCL12. Cancers.

[35] Tomiyama A., Kobayashi T., Mori K., Ichimura K. (2019). Protein Phosphatases—A Touchy Enemy in the Battle Against Glioblastomas: A Review. Cancers.

[36] Fahey J., Girotti A. (2019). Nitric Oxide Antagonism to Anti-Glioblastoma Photodynamic Therapy: Mitigation by Inhibitors of Nitric Oxide Generation. Cancers.

[37] Martikainen M., Essand M. (2019). Virus-Based Immunotherapy of Glioblastoma. Cancers.

[38] Stepanenko A., Chekhonin V. (2018). Recent Advances in Oncolytic Virotherapy and Immunotherapy for Glioblastoma: A Glimmer of Hope in the Search for an Effective Therapy?. Cancers.

[39] Fabian D., Guillermo Prieto Eibl M., Alnahhas I., Sebastian N., Giglio P., Puduvalli V., Gonzalez J., Palmer J. (2019). Treatment of Glioblastoma (GBM) with the Addition of Tumor-Treating Fields (TTF): A Review. Cancers.

[40] Lohmann P., Werner J., Shah N., Fink G., Langen K., Galldiks N. (2019). Combined Amino Acid Positron Emission Tomography and Advanced Magnetic Resonance Imaging in Glioma Patients. Cancers.

[41] Ahmad F., Sun Q., Patel D., Stommel J. (2019). Cholesterol Metabolism: A Potential Therapeutic Target in Glioblastoma. Cancers.

[42] Rominiyi O., Al-Tamimi Y., Collis S. (2019). The ‘Ins and Outs’ of Early Preclinical Models for Brain Tumor Research: Are They Valuable and Have We Been Doing It Wrong?. Cancers.

[43] Gao Y., Vallentgoed W., French P. (2018). Finding the Right Way to Target EGFR in Glioblastomas; Lessons from Lung Adenocarcinomas. Cancers.

